# Bacteraemia and Associated Complications on Imaging as a Clue to Colorectal Malignancy

**DOI:** 10.5334/jbsr.2855

**Published:** 2022-09-13

**Authors:** Jan Van Offenwert, Patrick Gillardin

**Affiliations:** 1 Ziekenhuis Oost-Limburg, BE

**Keywords:** Streptococcus gallolyticus, bacteraemia, colorectal malignancy, rectal tumour, pseudo-aneurysm

## Abstract

Streptococcus gallolyticus (SG) – among other bacterial infections – is associated with colorectal malignancy and adenoma. It is reported that patients with SG bacteraemia have a concomitant colorectal tumour in 25–80% of cases. We present a case of a patient with complications of this septicaemia associated with a rectal adenocarcinoma, as an example of this important radiological and clinical correlation.

**Teaching Point:** Always screen for primary colorectal malignancy in a patient with malignancy-associated bacterial infection.

## Case Report

An 81-year-old male patient was admitted to the hospital with fever and weight loss. Lab results showed merely signs of inflammation with elevated C-reactive protein (CRP) (96 mg/L) and white blood cell count (15.2 × 10^3^/µL). Haemocultures were taken and analysed over the following days.

**Figure 1:**
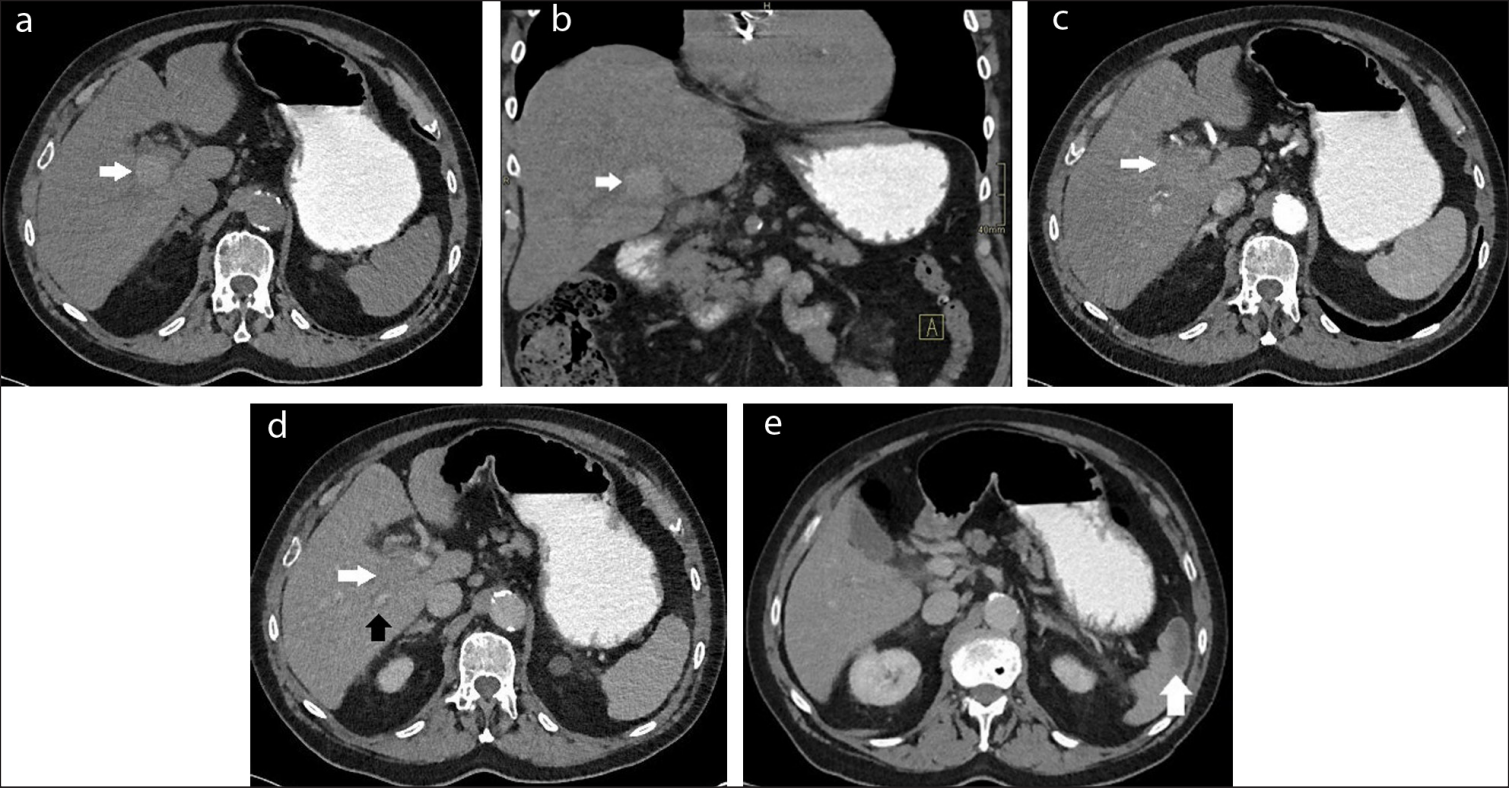
Axial **(a)** and coronal **(b)** unenhanced CT of the upper abdomen shows a homogenous spontaneous hyperdense ovoid lesion of 3.2 cm (white arrow). The arterial **(c)** and venous **(d)** phase of the contrast enhanced CT shows no enhancement, resulting in hypodense appearance relative to the liver. Compression of the right portal vein (d, black arrow) is depicted. In addition, a segmental splenic infarction is seen (**e**, white arrow).

**Figure 2:**
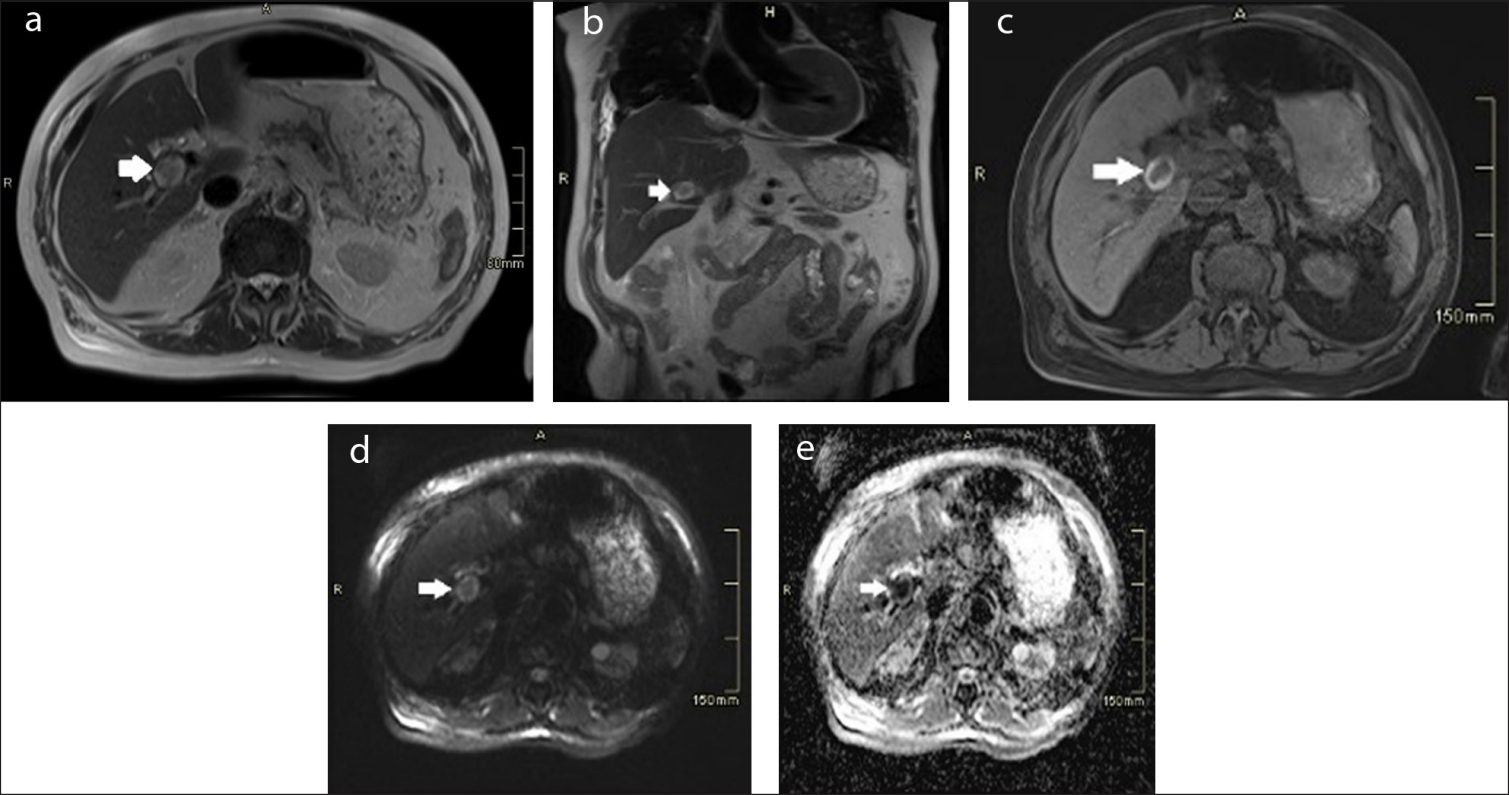
Axial **(a)** and coronal **(b)** fat-sat T2 and axial T1 **(c)** sequence show a lesion with a hyperintense periphery and a hypo-intense centre (white arrows). High signal intensity on the DWI **(d)** and low signal on ADC **(e)** is noted, confirming diffusion restrictive lesion (white arrows).

**Figure 3:**
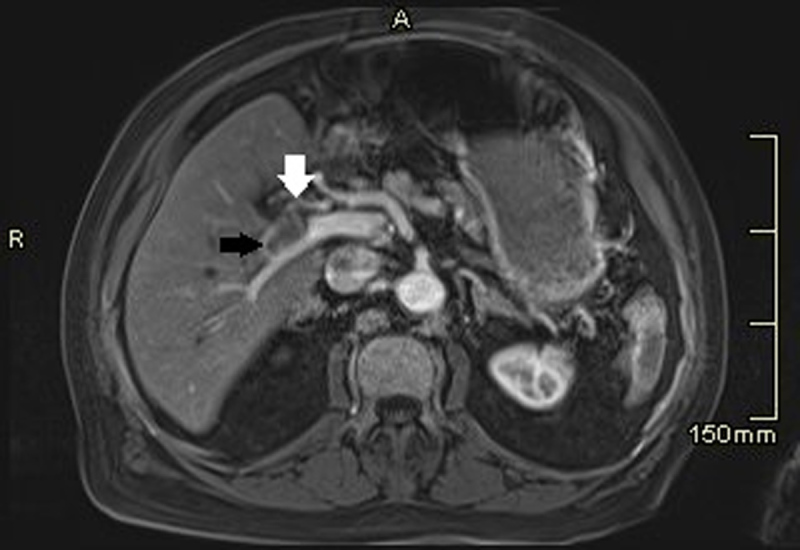
The arterial phase MRI shows this structure (partly visualised; black arrow) deriving from the right hepatic artery (white arrow).

**Figure 4:**
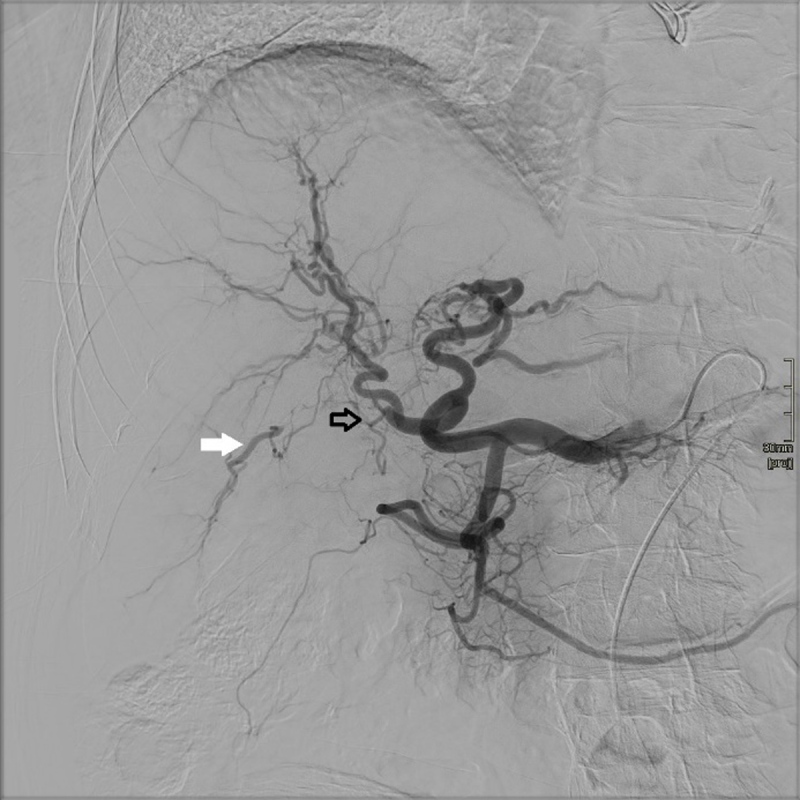
On this DSA image, the black hollow arrow indicates occlusion of the right hepatic artery, and re-injection is denoted by the white arrow.

The plain abdominal CT showed a homogenous spontaneously hyperdense nodule of 3.2 cm in liver segment 4 near the hilum (Figure 1). Furthermore, a segmental infarction of the spleen was seen (Figure 1). For further characterization of the liver lesion magnetic resonance imaging (MRI) was performed, which showed an ovoid lesion centred on the right portal vein pedicle. The lesion had a fat-sat T1-weighted and T2-weighted hyperintense periphery and T1-weighted and T2-weighted hypo-intense centre with significant diffusion restriction and low Apparent diffusion coefficient (ADC) values (Figure 2). An occlusion of the right hepatic artery extending into the lesion was suspected (Figure 3), which could not be documented on prior imaging performed months earlier. These clinical and imaging findings are highly suggestive of a (mycotic) thrombosed pseudo-aneurysm of the right hepatic artery (Figure 3). Digital subtraction angiography (DSA) confirmed the occlusion of the right proximal hepatic artery (Figure 4).

**Figure 5:**
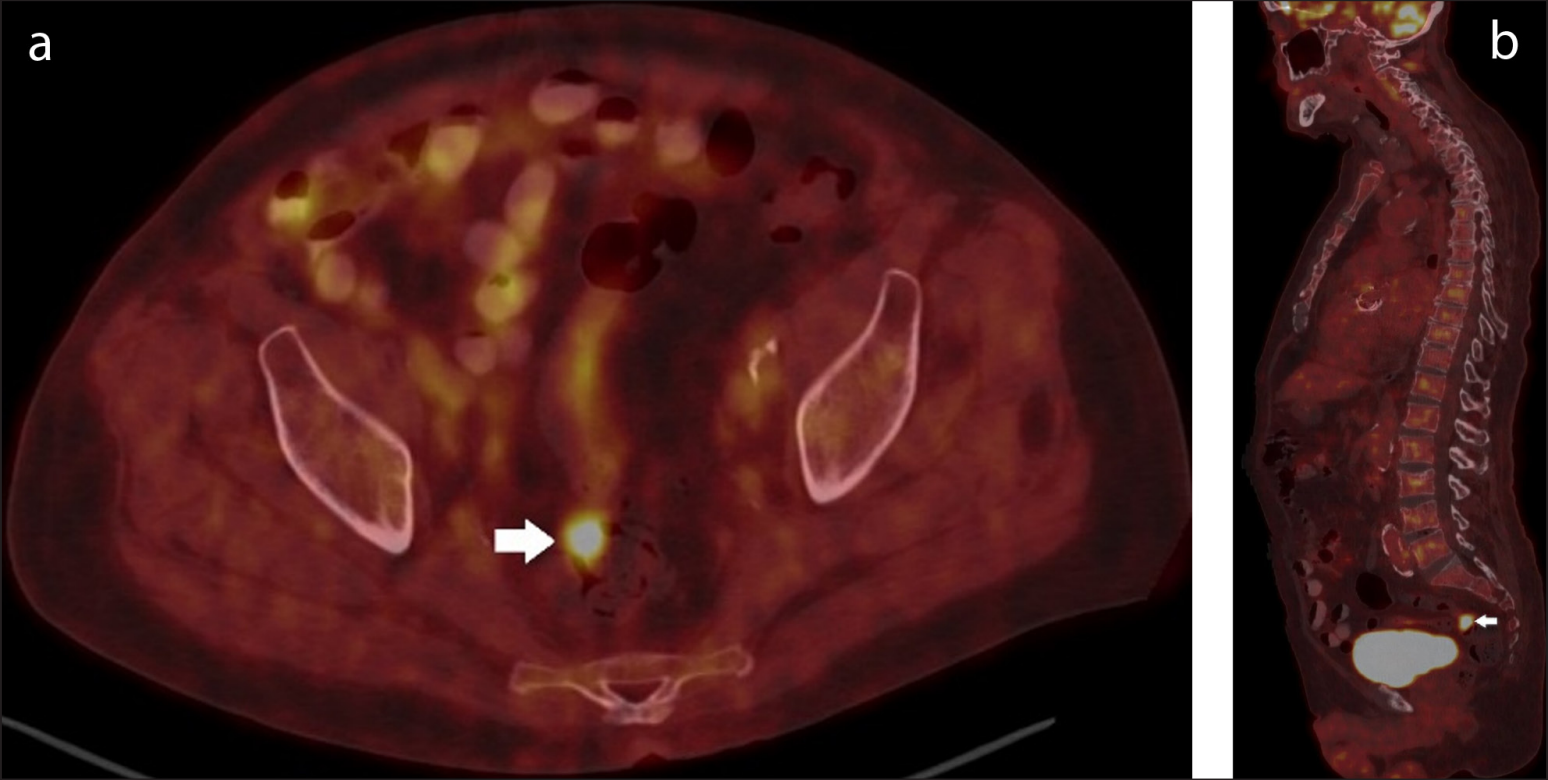
The axial **(a)** and sagittal **(b)** PET-CT shows avid FDG-uptake in a lesion in the rectum (white arrows).

Just before the scheduled MRI, the result of the haemocultures revealed S. gallolyticus (SG) bacteraemia. Based on the observations on the MRI, the radiologists told the referral physician that screening colorectal malignancy would be mandatory. Positron Emission Tomography-Computed Tomography (PET-CT) was performed to locate the source of infection and screen for malignancy. A focus of F-18 fluorodeoxyglucose-uptake (FDG-uptake) was seen in the rectal wall (Figure 5), suggestive of malignancy. Other than uptake at the site of the suspected (mycotic) thrombosed pseudo-aneurysm, no other site of infection or potential malignancy was observed. At the time of presentation, the patient did not have any gastro-intestinal complaints and previous faecal occult blood tests (iFOBT) for colorectal cancer screening all were negative.

**Figure 6:**
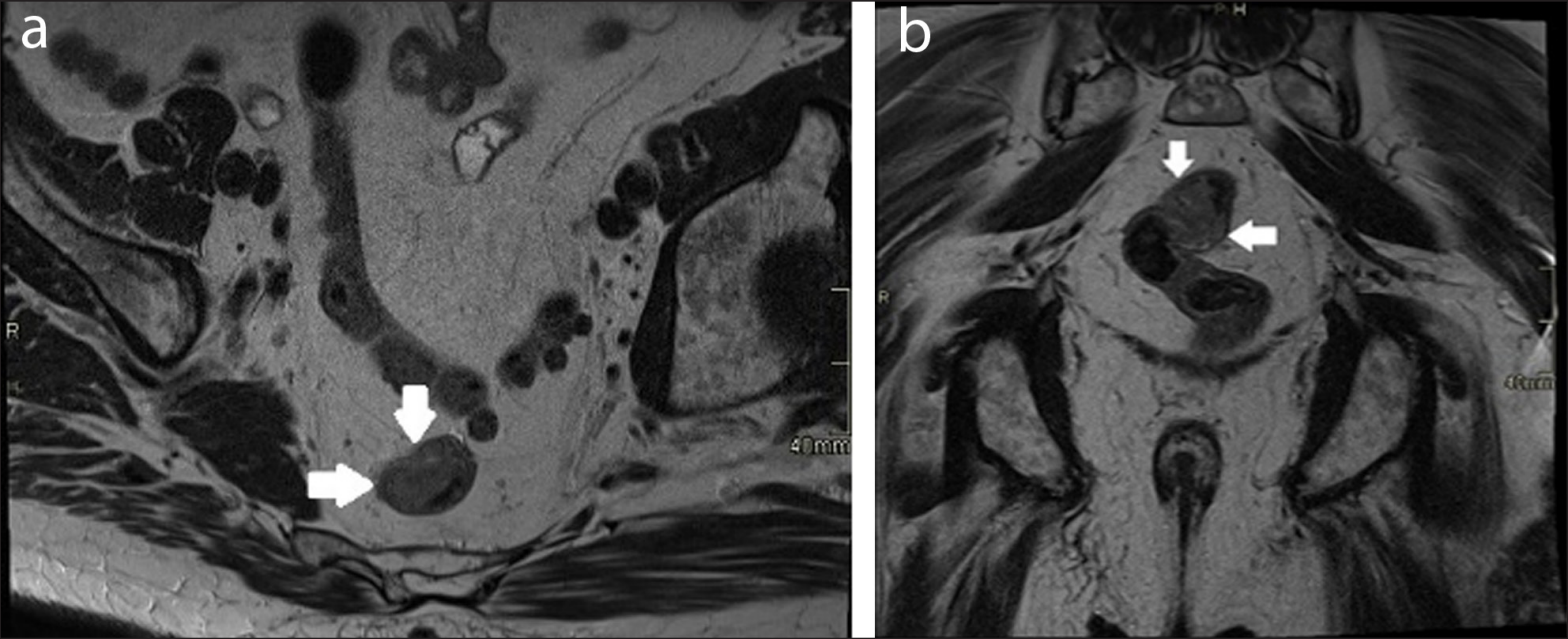
Axial **(a)** and coronal **(b)** T2-weighted sequence of the lower pelvis, showing a iso-intense semi-circular, sessile mass in the middle 1/3 of the rectum (white arrows).

MRI of the rectum (Figure 6) confirmed a tumour of the rectal wall, which was already described on colonoscopy. After successful surgery, the histological diagnosis was an invasive, well differentiated adenocarcinoma, staged as pT1N0M0.

## Discussion

Streptococcus gallolyticus (SG) is part of the group of Streptococcus bovis pathogens and was previously known as S. bovis biotype I [1]. SG has been associated with colorectal malignancy and adenoma [2]. A few years ago, SG was detected in 49% of colorectal cancer tissue, while only in 8% of colonic samples of healthy patients, which clearly correlates with increased faecal carriage of SG in patients with colorectal malignancy [2]. It has been reported that patients with bacteraemia caused by SG have a concomitant colorectal tumour in 25–80% of cases [1]. Besides S. gallolyticus, other well-studied bacterial infections that might play a role in carcinogenesis include B. fragilis, F. nucleatum, P. species, C. septicum, C. perfringens, and G. morbillorum [3].

Although the pathophysiology of SG is not fully understood yet, it is hypothesised that the carcinoma is the entry site of the pathogens to the bloodstream [2]. Many of these malignancy-associated pathogens are commonly part of the colonic microbiome, and therefore, occurrence in the bloodstream suggests migration through a damaged bowel wall as in neoplasia [3]. It has been demonstrated that some of these bacteria preferably settle in neoplastic lesions accompanied by chronic inflammation, inducing vasodilatation and facilitating permeability of the neoplastic capillaries, thus causing bacteraemia [3].

This correlation could explain recurrent episodes of SG bacteraemia sensitive to appropriate antibiotic therapy, as in our case. After rectal surgery, no recurrent bacteremia was reported.

## Conclusion

This case demonstrates that knowledge of the association of S. bovis/S. gallolyticus with (colo)rectal malignancy contributes to the early detection of malignant lesions and therefore possible improved patient outcome. Additional diagnostic examinations (MRI, PET-CT and/or colonoscopy) are recommended to exclude concomitant colorectal malignancy.
